# Six-year survival and clinical performance of glass hybrid restorations following selective caries removal in teeth with molar incisor hypomineralization: a prospective cohort study

**DOI:** 10.1007/s00784-025-06358-6

**Published:** 2025-05-05

**Authors:** Berkant Sezer, Betül Şen Yavuz, Ceyda İpek İşseven, Nihan Tuğcu, Cansu Çalışkan, Başak Durmuş, Betül Kargül

**Affiliations:** 1 Department of Pediatric Dentistry, School of Dentistry, Çanakkale Onsekiz Mart University, Çanakkale, Türkiye; 2 Department of Pediatric Dentistry, School of Dentistry, Marmara University, Istanbul, Türkiye; 3 Private Practice, Istanbul, Türkiye; 4 Queen Mary University of London, London, UK

**Keywords:** Molar incisor hypomineralization, Selective caries removal, Survival analysis, Glass hybrid restorations, Developmental dental anomalies

## Abstract

**Objectives:**

This study aimed to evaluate the clinical success and long-term survival of glass hybrid restorations in permanent first molars affected by molar incisor hypomineralization (MIH) following selective caries removal (SCR) over a six-year follow-up period.

**Materials and methods:**

This prospective cohort study included a total of 134 MIH-affected molars in 58 children (mean age 8.94 ± 1.41 years) restored with glass hybrid materials after SCR. Clinical outcomes were assessed at baseline and at 6, 12, 18, 24, and 72 months using modified USPHS criteria. Kaplan-Meier survival analysis and Cox proportional hazards regression with robust standard errors were conducted to evaluate restoration survival and identify potential predictors.

**Results:**

The overall estimated mean survival time of restorations was 59.82 ± 1.50 months. The survival probabilities for mild and severe lesions at six-year were 24.3% and 11.1%, respectively. Multivariate Cox proportional hazards regression with robust standard errors indicated that lesion severity, medium lesion extension, and large lesion extension had a statistically significant impact on restoration survival (*p* < 0.001 for all). While survival was satisfactory up to 2–3 years, a marked decline was observed over time, particularly in severely affected molars.

**Conclusions:**

Glass hybrid restorations demonstrated acceptable clinical performance in MIH-affected molars in the short to medium term but showed significant limitations in long-term survival.

**Clinical relevance:**

The need for advancements in restorative materials tailored to the structural challenges of MIH. Glass hybrid materials remain a viable option for managing MIH-affected molars, particularly in young patients where minimally invasive approaches are prioritized.

## Introduction

Molar incisor hypomineralization (MIH), a developmental enamel defect, first emerged with various definitions in the 1970s and was officially characterized in 2001 as a qualitative enamel defect with a hypomineralized nature. It typically affects at least one permanent first molar and often involves permanent incisors [1]. From an epidemiological perspective, MIH is the most prevalent enamel defect, and its reported prevalence has been increasing since its initial definition [1]. Globally, MIH affects approximately 12.8% of the population [95% Confidence Interval (CI): 11.5–14.1%], equating to around 878 million individuals [2]. Each year, an average of 17.5 million new cases are identified, with 27.4% requiring treatment for issues such as pain, hypersensitivity, caries, aesthetic concerns, and post-eruptive enamel breakdown [3]. The etiology of MIH remains uncertain; however, pre-, peri-, and post-natal factors, along with genetic, epigenetic, and environmental influences, are increasingly recognized as potential contributors. Ongoing research continues to explore the roles of these factors in the development of the condition [4].

The symptoms observed in teeth affected by MIH vary depending on the severity of the condition and generally range from mild to severe. These include white/creamy and yellow/brown demarcated opacities, post-eruptive enamel breakdown, atypical caries, and restorations, and, in severe cases, tooth loss [5]. Due to the compromised enamel structure and poor oral hygiene, toothaches may progress from reversible to irreversible pulpitis because of the rapid and aggressive progression of existing caries [6]. Chronic inflammation caused by continuous bacterial invasion from porous enamel to dentin can make achieving adequate anesthesia challenging, often leading to behavioral issues during dental treatment [7]. Moreover, aesthetic concerns and a significant decline in the individual’s quality of life are common outcomes due to the cumulative effects of these factors [8].

The rapid and aggressive progression of caries in MIH-affected teeth highlights the importance of conservative treatment approaches to minimize the risk of pulp exposure and postoperative sensitivity during restorations [9]. Selective caries removal (SCR) is particularly beneficial for deep carious lesions, as it involves removing softened dentin at the cavity margins while preserving leathery dentin near the pulp. This technique reduces the need for endodontic treatment [10]. In SCR, achieving complete sealing at the margins deprives bacteria within the lesion of carbohydrate sources, effectively halting its progression [10].

Various materials, including amalgam, glass ionomers, composites, glass hybrids, preformed metal crowns, and indirect restorations, have been utilized in the management of MIH-affected molars [9, 11]. However, clinical challenges such as the high risk of pulp exposure, the immediate fracture susceptibility of highly porous enamel with low hardness and elastic modulus and increased postoperative sensitivity have driven ongoing investigations into conservative approaches, including direct restorations and SCR [11, 12].

The hydrophilic nature of glass ionomers, coupled with their fluoride-releasing capability, offers distinct advantages in SCR for MIH-affected teeth [11, 12]. However, the low mechanical properties of traditional glass ionomers have limited their use. In contrast, newly developed glass hybrid materials exhibit improved wear resistance and hardness, prompting increased interest in their application for MIH management [9, 11–13]. Despite this, no studies to date have reported the long-term survival of restorations using glass hybrid materials.

Thus, the aim of this study was to evaluate the survival and clinical performance of MIH-affected molars restored with glass hybrids following SCR. The primary null hypothesis was that there would be no significant difference in the overall survival of restorations between molars affected by mild and severe MIH. The secondary null hypothesis was that there would be no statistically significant differences in restoration satisfaction across the relevant categories.

## Materials and methods

### Study characteristics and design

This study adhered to the Strengthening the Reporting of Observational Studies in Epidemiology (STROBE) guidelines and was conducted in accordance with the ethical principles of the Helsinki Declaration for medical research involving human subjects. Ethical approval for the study protocol was obtained from the Clinical Research Ethics Committee of the School of Dentistry, Marmara University, Istanbul, Türkiye (Approval No. 569123). Written informed consent was obtained from the parents or guardians of all participants, following the assent of the children. This prospective cohort study was conducted at the Pediatric Dentistry Clinic of the School of Dentistry, Marmara University.

### Sample size calculation

The sample size calculation was based on a study by Klinke et al. [14], which assessed the clinical performance of glass hybrid restorations in various cavity sizes. Using the G*Power Version 3.1.9.6 program (Universitat Kiel, Kiel, Germany), the minimum sample size was calculated to be 105, with a 95% confidence level (1-α), 85% test power (1-β), and an effect size of d = 0.371.

### Study population, inclusion, and exclusion criteria

The intraoral examinations of children aged 8–11 who visited the Pediatric Dentistry Clinic at the School of Dentistry, Marmara University, for routine check-ups between June 2016 and June 2017 were conducted under standardized conditions. Examinations were performed in the same dental chair, under consistent reflector light, using a flat-surfaced no. 4 mouth mirror. Both hard and soft tissues were assessed in a wet state, with drying achieved using cotton rolls and air spray when necessary to confirm visual diagnoses.

The diagnosis of MIH followed the guidelines of the European Academy of Paediatric Dentistry (EAPD) [15], which stipulate that a lesion must be at least 2 mm in diameter to qualify for an MIH diagnosis. Lesions smaller than 2 mm were excluded. According to these guidelines, teeth affected by MIH may present with demarcated white/creamy and/or yellow/brown opacities, post-eruptive enamel breakdown, atypical restorations, atypical caries, or tooth loss due to MIH. At least one of these features in a permanent first molar is required for an MIH diagnosis [15].

Examinations were performed by a single experienced pediatric dentist with the supervisor of the study. Before the main study, the pediatric dentist responsible for diagnosis was trained on the diagnostic criteria for MIH using intraoral photographs of 40 patients. Subsequently, the study supervisor calibrated the examiner for MIH diagnosis using 20 patients who were not included in the study. Ten days later, the examiner re-evaluated the same patients independently, and the intra-examiner Kappa value for MIH diagnosis was calculated as 0.97.

#### Inclusion criteria

• Fully erupted teeth or those with an occlusal surface entirely visible in the oral cavity. • No clinical or radiographic evidence of spontaneous pain, fistula, abscess, or other signs of irreversible pulpitis or pulp necrosis. • Atypical caries associated with MIH defects [MIH Treatment Need Index (TNI) 2a–c] [16]. • Periodontally healthy teeth. • Sufficient patient cooperation for restorative treatment. • Written informed consent obtained from the parent/guardian of participant.

#### Exclusion criteria

• Teeth with developmental enamel defects other than MIH. • Children unlikely to attend recall appointments. • Children with developmental systemic anomalies, bruxism, orthodontic appliances, or allergies to restorative materials used. • Molars with restorations, fractures, cracks, erosive or abrasive tooth wear, spontaneous pain, or pulp involvement. • Teeth requiring local anesthesia during the clinical procedure (local anesthesia was not applied due to difficulties in achieving effective anesthesia in MIH-affected teeth, which often exhibit chronic inflammation due to defective enamel). This standardization aimed to avoid confounding factors by including only teeth without pulp involvement (such as irreversible pulpitis or pulp necrosis) and ensuring consistency in the treatment protocol.

The severity and extent of the lesions were categorized using the Würzburg MIH concept [16]. According to this classification: • Defects covering less than one-third of the tooth (2a) were classified as mild in severity and small (S) in extension. • Defects involving one-third to two-thirds of the tooth were classified as mild in severity and medium (M) in extension. • Defects affecting more than two-thirds of the tooth or located close to the pulp were categorized as severe in severity and large (L) in extension.

### Clinical procedures

All clinical procedures were performed by a single pediatric dentist. Defective enamel affected by MIH was removed without local anesthesia using diamond round burs (Meisinger Dental Burs, Hager & Meisinger GmbH, Neuss, Germany) attached to an air-turbine handpiece (NSK, Japan), exposing the underlying carious dentin. The hypomineralized enamel was selectively removed, targeting the enamel with an opaque appearance that disintegrates upon contact with instruments, continuing until more durable enamel was reached. This selective removal was carefully carried out to avoid excessive loss of healthy enamel, ensuring optimal restoration margins. Further excavation of the carious tissue was carried out with tungsten carbide burs (Meisinger Dental Burs) attached to a low-speed handpiece (NSK) until firm and dry dentin was reached. To ensure proper adaptation of the restoration margins, all caries were removed peripherally, including the enamel-dentin junction, while preserving as much healthy enamel as possible to support the restoration. The hardness of the dentin was periodically assessed with a blunt-ended dental probe. For pulpal-axial caries, the carious dentin was carefully removed using a sharp excavator without applying excessive force, adhering to the principle of SCR to avoid pulp exposure. Ultimately, slightly moist, leather-like dentin was left intact to minimize the risk of pulpal perforation while following the principles of SCR.

Glass hybrid restorations were applied according to the manufacturer’s instructions. Isolation of the tooth was achieved using cotton rolls and a saliva ejector. The cavity was conditioned with Cavity Conditioner^®^ (GC Europe, Leuven, Belgium) for 10 s, rinsed with water, and isolated again using a cotton roll before being dried with dry cotton pellets. While maintaining a dry working field, the operator activated the glass hybrid restorative material (Equia Forte^®^, GC Europe) in a manufacturer-recommended mixer for 10 s. The activated material, contained in a capsule, was immediately loaded into an applicator, and applied to the cavity. The material was compressed for 40 s using a finger coated with petroleum jelly, and excess material was carefully removed with a dental carver. After a setting time of 2.5 min, the occlusion was verified with fine carbon paper. The restoration surface was cleaned with dry cotton pellets, followed by the application of a resin-based light-cured surface sealant (Equia Coat^®^, GC Europe). The sealant was cured for 20 s using a light-curing device (D-Light Duo, GC Europe, 1,400 mW/cm²), completing the procedure. Postoperative instructions included advising the child to avoid eating or drinking for minimum one hour and to minimize chewing on the side of the treated tooth for the remainder of the day.

### Evaluation

The restorations were evaluated using modified United States Public Health Service (USPHS) criteria [17]. According to these criteria, the restored teeth and restorations were assessed during follow-up sessions for anatomical form, marginal adaptation, surface texture, marginal discoloration, retention, secondary caries, and postoperative sensitivity. Evaluations were conducted by an experienced pediatric dentist who was not involved in the diagnosis or treatment and was blinded to the study details. To ensure intra-examiner reliability, 15 randomly selected restorations were re-evaluated two weeks apart, yielding excellent reliability with a Cohen’s Kappa coefficient of 0.93.

**Fig. 1:**
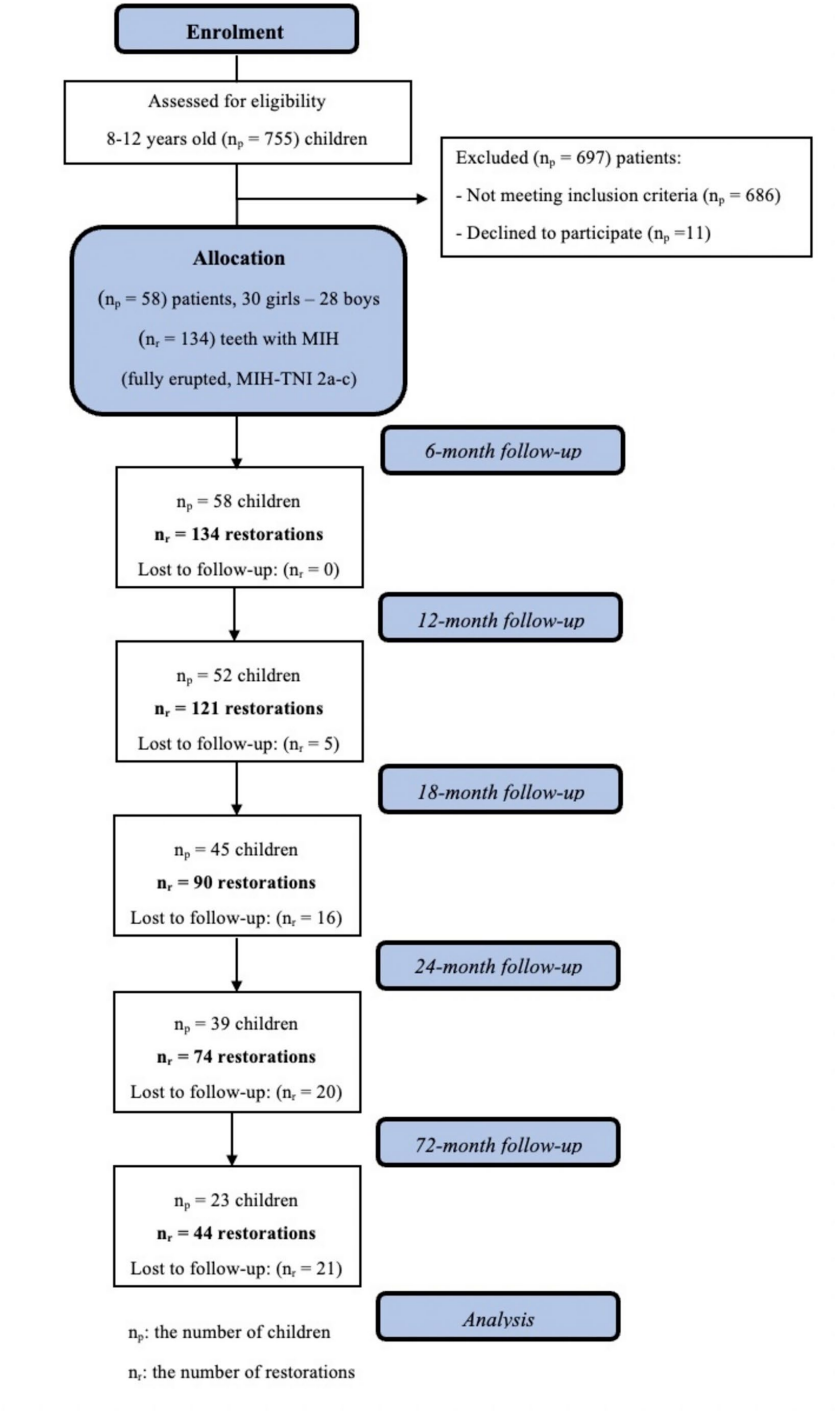
CONSORT flow chart for the progress of the study

**Fig. 2:**
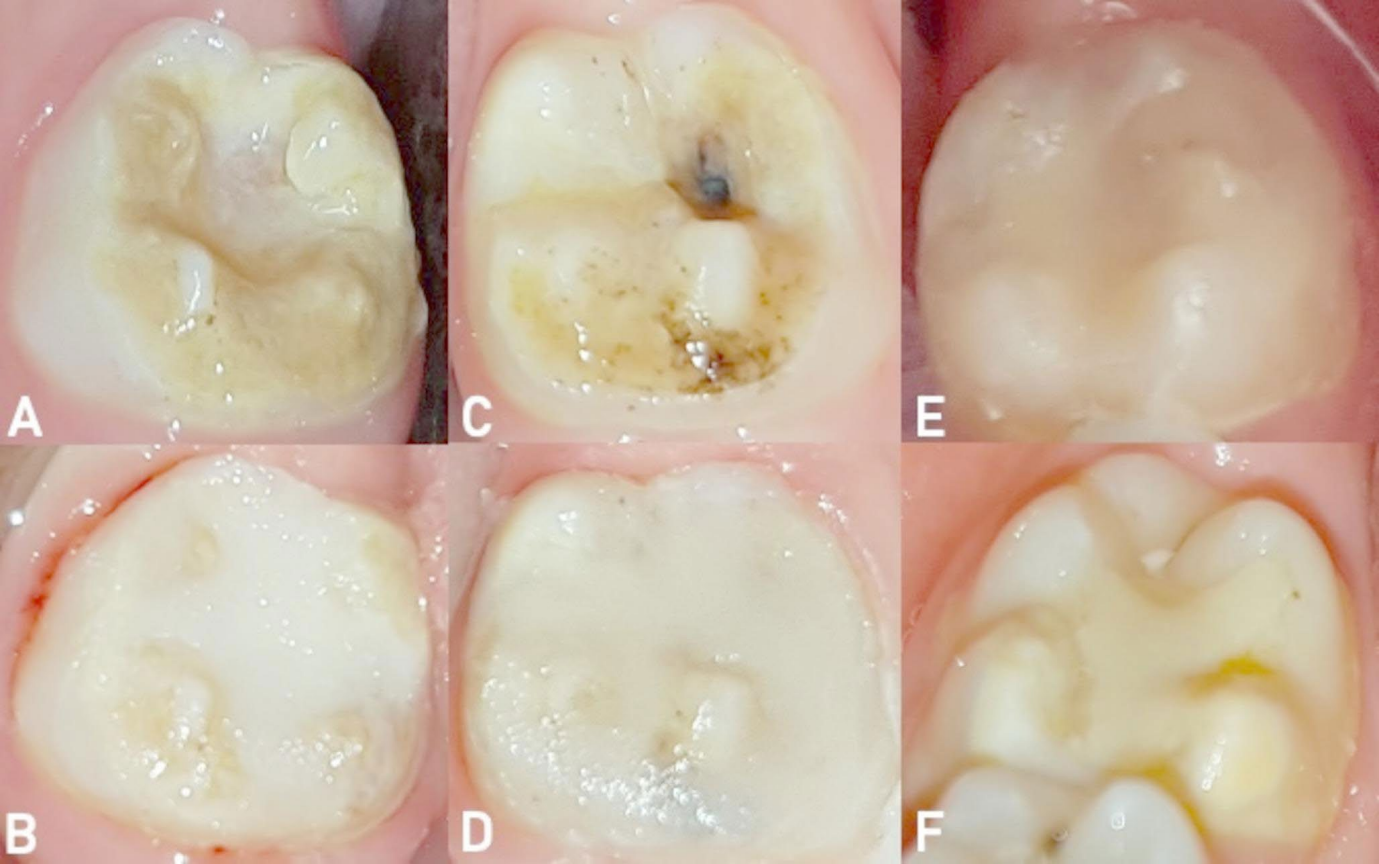
Examples of clinical intraoral images of glass hybrid restorations and follow-up of first permanent molars affected by molar incisor hypomineralization. A–B. Pre-operative and 24-month follow-up images (scored as alpha in terms of all criteria) of the restoration of the mandibular right permanent first molar, C–D. Pre-operative and 24-month follow-up images (scored as alpha in terms of all criteria) of the mandibular left permanent first molar, E. Six-year image (scored as bravo in terms of marginal discoloration) of the restoration of the mandibular left permanent first molar, F. 24-month image (scored as bravo in terms of retention and charlie in terms of post-operative sensitivity) of the restoration of the mandibular right permanent first molar

All evaluations were performed under standardized conditions in the same dental unit, with adequate reflector lighting, using a dental probe and a flat-surfaced mouth mirror. Follow-up assessments were conducted at 6, 12, 18, 24, and 72 months, as depicted in the flow chart (Fig. 1). Figure 2 illustrates the MIH-affected molars, the glass hybrid restorations applied to these teeth, and the intraoral clinical photographs taken during the follow-up sessions.

According to the modified USPHS criteria, the presence of secondary caries or postoperative sensitivity was recorded as “Charlie,” while their absence was recorded as “Alpha.” For other criteria, scores of “Alpha,” “Bravo,” and “Charlie” were used, with “Alpha” and “Bravo” categorized as “satisfied.” A restoration was deemed “unsatisfied” if it received a “Charlie” score for any criterion, marking the endpoint for that restoration in the study. Specifically, “Charlie” scores indicating severe marginal degradation, complete loss of anatomical form, extensive discoloration, or loss of retention were classified as failures. Minor marginal discoloration or slight anatomical changes (scored as “Bravo”) were considered clinically acceptable and did not contribute to failure classification. In cases of restorative failure without additional adverse symptoms, repair or replacement of the restoration was planned. However, if spontaneous pain, fistula, or abscess was observed, the affected tooth was designated for endodontic treatment or extraction, and the treatment plan was adjusted accordingly.

### Statistical analysis

Mean and standard deviation were calculated for age, decayed-filled teeth (dft), and decayed, missing, and filled teeth (DMFT) variables. Descriptive statistics were presented as frequencies and percentages. Relationships between categorical variables were analyzed using the Chi-Square (χ²) test. Missing data due to loss to follow-up were addressed using a complete-case analysis approach. Only participants with available data at each follow-up point were included in the respective analyses, and no data imputation was performed. The Kaplan-Meier survival analysis, employing a Mantel-Cox log-rank model with 95% CIs, was used to evaluate the survival time for each group. Differences between survival curves were determined using the log-rank test. The independent variables of the arch of the treated molar, lesion extension, lesion severity, and categorical age of the child were modeled using a Cox proportional hazards regression model. To account for the potential intra-subject dependency arising from multiple restorations per child, robust standard errors were estimated in the Cox proportional hazards regression model. The significance of individual predictors was evaluated based on the Wald test, and the hazard ratios with 95% CIs were reported. Data analysis was conducted using SPSS version 26.0 (IBM Corporation, Chicago, Illinois, USA), with a significance level set at 0.05.

## Results

In this study, 134 fully erupted MIH-affected molars from 58 children (30 girls, 28 boys) were restored with glass hybrid after SCR. The mean age of the children was 8.94 years (± 1.41). At baseline, the mean dft and DMFT values were 3.96 (± 3.46) and 3.63 (± 1.44), respectively. Loss to follow-up at 6, 12, 18, 24, and 72 months was 0, 5, 16, 20, and 21, respectively.

Table 1 presents the modified USPHS criteria and the satisfactory status of restorations at all follow-up points. A total of 71 restorations were classified as unsatisfactory by the six-year follow-up. Of these, 37 restorations were scored “Charlie” for anatomical form, 43 for marginal adaptation, 47 for retention, 46 for secondary caries, 42 for surface texture, 40 for marginal discoloration, 42 for color match, and 40 for postoperative sensitivity. More than half of the “Charlie” scores for all criteria, except for postoperative sensitivity (which was nearly one-third), were recorded during the six-year follow-up evaluation. Additionally, half of the unsatisfactory restorations were observed at the six-year follow-up. Moreover, over the six-year period, 15 restorations (1 mild, 14 severe) were considered failures solely due to receiving a “Charlie” score in the postoperative sensitivity criterion, despite achieving an “Alpha” score in all other criteria.

**Table 1:** Results of evaluation of restorations according to modified USPHS criteria and satisfaction status at 6, 12-, 18-, 24- and 72-months follow-up

Modified USPHS Criteria	Baseline (*N* = 134)	6 months (*N* = 134)	12 months (*N* = 121)	18 months (*N* = 90)	24 months (*N* = 74)	72 months (*N* = 44)
Anatomical form N (%)						
A	134 (100)	121 (90.3)	102 (84.3)	80 (88.9)	53 (71.6)	10 (22.7)
B	0	13 (9.7)	10 (8.3)	8 (8.9)	16 (21.6)	13 (29.5)
C	0	0	9 (7.4)	2 (2.2)	5 (6.8)	21 (47.7)
Marginal adaptation N (%)						
A	134 (100)	122 (91)	99 (81.8)	77 (85.6)	50 (67.6)	5 (11.4)
B	0	10 (7.5)	12 (9.9)	11 (12.2)	20 (27)	14 (31.8)
C	0	2 (1.5)	10 (8.3)	2 (2.2)	4 (5.4)	25 (56.8)
Retention N (%)						
A	134 (100)	129 (96.3)	110 (90.9)	85 (94.4)	64 (86.5)	8 (18.2)
B	0	5 (3.7)	4 (3.3)	3 (3.3)	3 (4.1)	5 (11.4)
C	0	0	7 (5.8)	2 (7)	7 (9.5)	31 (70.5)
Secondary caries N (%)						
A	134 (100)	131 (97.8)	113 (93.4)	88 (97.8)	68 (91.9)	17 (38.6)
C	0	3 (2.2)	8 (6.6)	2 (2.2)	6 (8.1)	27 (61.4)
Surface texture N (%)						
A	134 (100)	130 (97)	110 (90.9)	84 (93.3)	57 (77)	11 (25)
B	0	4 (3)	5 (4.1)	4 (4.4)	11 (14.9)	5 (11.4)
C	0	0	6 (5)	2 (2.2)	6 (8.1)	28 (63.6)
Marginal discoloration N (%)						
A	134 (100)	127 (94.8)	103 (85.1)	79 (87.8)	55 (74.3)	11 (25)
B	0	7 (52.)	11 (9.1)	9 (10)	13 (17.6)	8 (18.2)
C	0	0	7 (5.8)	2 (2.2)	6 (8.1)	25 (56.8)
Color match N (%)						
A	134 (100)	126 (94)	107 (88.4)	84 (93.3)	63 (85.1)	9 (20.5)
B	0	8 (6)	6 (5)	4 (4.4)	5 (6.8)	9 (20.5)
C	0	0	8 (6.6)	2 (2.2)	6 (8.1)	26 (59.1)
Postoperative sensitivity N (%)						
A	134 (100)	129 (96.3)	109 (90.1)	86 (95.6)	70 (94.6)	29 (65.9)
C	0	5 (3.7)	12 (9.9)	4 (4.4)	4 (5.4)	15 (34.1)
Satisfactory status N (%)						
Satisfied	134 (100)	126 (94)	106 (87.6)	86 (95.6)	65 (87.8)	10 (22.2)
Unsatisfied	0	8 (6)	15 (12.4)	4 (4.4)	9 (12.2)	35 (77.8)
*N: Number of restorations; A: Alpha score evaluated as “successful*,*” B: Bravo score evaluated as “acceptable*,*” C: Charlie score evaluated as “unsuccessful” from modified USPHS criteria.*						

The comparison results of restoration satisfaction across the child’s age, the arch of the treated molar, the severity of the lesion, and the extent of the lesion categories are presented in Table 2. None of the categories showed a statistically significant difference in restoration satisfaction (*p* > 0.05 for all categories at all time intervals).

**Table 2:** Restorations’ satisfaction status at different time points based on age, arch, lesion severity, and extension

	6 months					12 months					18 months					24 months					72 months					
Age	7–9 years		10–12 years		*p*-value	7–9 years		10–12 years		*p*-value	7–9 years		10–12 years		*p*-value	7–9 years		10–12 years		*p*-value	7–9 years		10–12 years		*p*-value	
Satisfied N (%)	84 (94.4)		42 (93.3)		1.000^§^	71 (85.5)		35 (92.1)		0.385^§^	54 (96.4)		32 (94.1)		0.631^§^	43 (87.8)		22 (88)		1.000^§^	5 (17.9)		5 (29.4)		0.467^§^	
Unsatisfied N (%)	5 (5.6)		3 (6.7)			12 (14.5)		3 (7.9)			2 (3.6)		2 (5.9)			6 (12.2)		3 (12)			23 (82.1)		12 (70.6)			
Total N (%)	89 (100)		45 (100)			83 (100)		38 (100)			56 (100)		34 (100)			49 (100)		25 (100)			28 (100)		17 (100)			
**Dental Arch**	**Upper**		**Lower**		**p-value**	**Upper**		**Lower**		**p-value**	**Upper**		**Lower**		**p-value**	**Upper**		**Lower**		**p-value**	**Upper**		**Lower**		**p-value**	
Satisfied N (%)	67 (97.1)		59 (90.8)		0.156^§^	57 (89.1)		49 (86)		0.606^†^	47 (94)		39 (97.5)		0.626^§^	37 (90.2)		28 (84.8)		0.501^§^	6 (24)		4 (20)		1.000^§^	
Unsatisfied N (%)	2 (2.9)		6 (9.2)			7 (10.9)		8 (14)			3 (6)		1 (2.5)			4 (9.8)		5 (15.2)			19 (76)		16 (80)			
Total N (%)	69 (100)		65 (100)			64 (100)		57 (100)			50 (100)		40 (100)			50 (100)		33 (100)			25 (100)		20 (100)			
**Lesion Severity**	**Mild**		**Severe**		**p-value**	**Mild**		**Severe**		**p-value**	**Mild**		**Severe**		**p-value**	**Mild**		**Severe**		**p-value**	**Mild**		**Severe**		**p-value**	
Satisfied N (%)	29 (96.7)		97 (93.3)		0.683^§^	27 (93.1)		79 (85.9)		0.518^§^	21 (91.3)		65 (97)		0.269^§^	15 (88.2)		50 (87.7)		1.000^§^	5 (35.7)		5 (16.1)		0.244^§^	
Unsatisfied N (%)	1 (3.3)		7 (6.7)			2 (6.9)		13 (14.1)			2 (8.7)		2 (3)			2 (11.8)		7 (12.3)			9 (64.3)		26 (83.9)			
Total N (%)	30 (100)		104 (100)			29 (100)		92 (100)			23 (100)		67 (100)			17 (100)		57 (100)			14 (100)		31 (100)			
**Lesion Extension**	**S**	**M**		**L**	**p-value**	**S**	**M**		**L**	**p-value**	**S**	**M**		**L**	**p-value**	**S**	**M**		**L**	**p-value**	**S**	**M**		**L**	**p-value**	
Satisfied N (%)	26 (96.3)	37 (97.4)		63 (91.3)	0.362^‡^	25 (96.2)	32 (88.9)		49 (83.1)	0.183^‡^	19 (86.4)	27 (100)		40 (97.6)	0.055^‡^	14 (93.3)	21 (84)		30 (88.2)	0.664^‡^	5 (38.5)	4 (22.2)		1 (7.1)	0.130^‡^	
Unsatisfied N (%)	1 (3.7)	1 (2.6)		6 (8.7)		1 (3.8)	4 (11.1)		10 (16.9)		3 (13.6)	0		1 (2.4)		1 (6.7)	4 (16)		4 (11.8)		8 (61.5)	14 (77.8)		13 (92.9)		
Total N (%)	27 (100)	38 (100)		69 (100)		26 (100)	36 (100)		59 (100)		22 (100)	27 (100)		41 (100)		15 (100)	25 (100)		34 (100)		13 (100)	18 (100)		14 (100)		
N: *Number of restorations*,* S: Small*,* M: Medium*,* L: Large.* †Pearson Chi-square test, §Fisher’s Exact test, ‡Likelihood ratio, *p* < 0.05																										

**Fig. 3:**
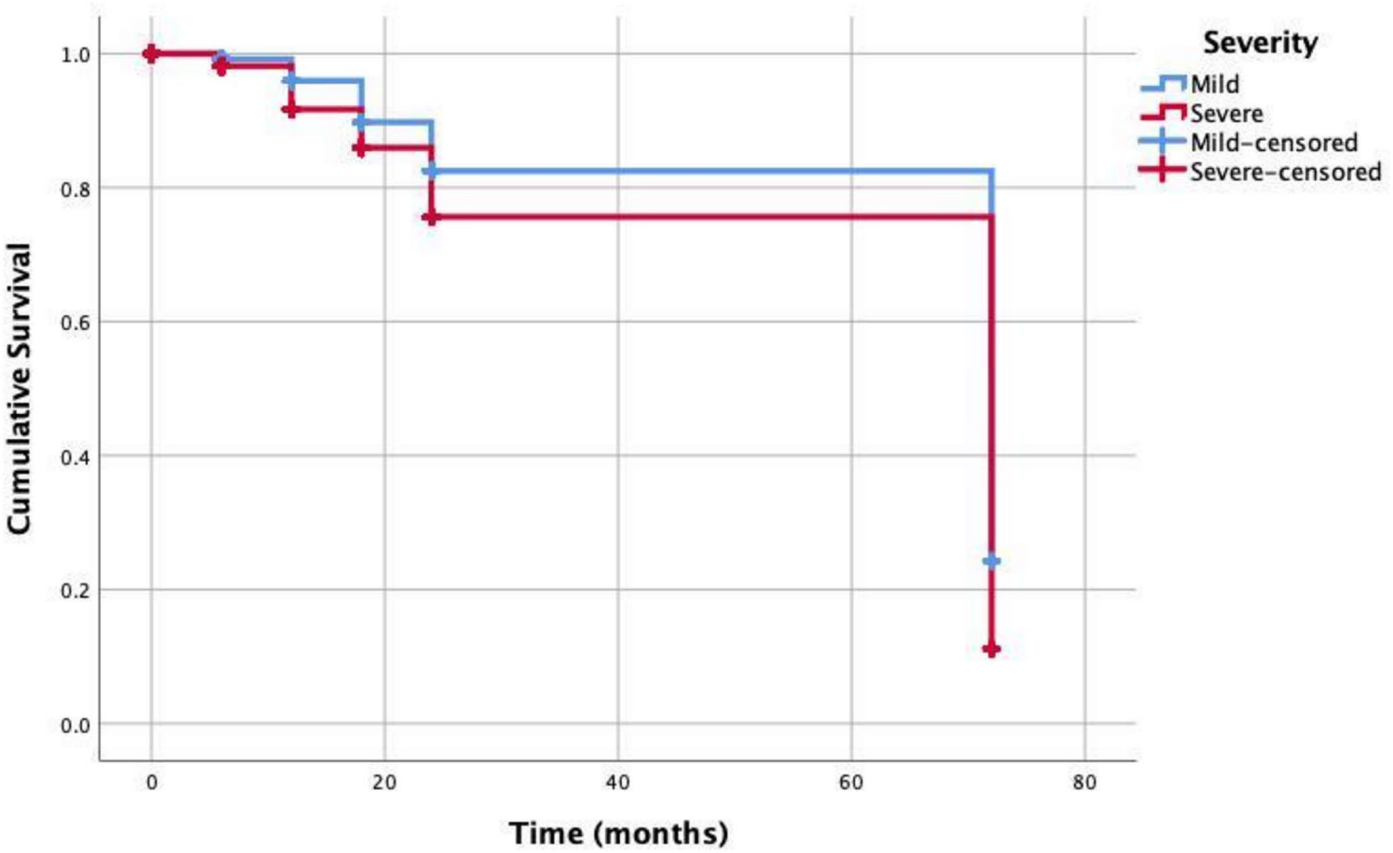
Kaplan-Meier survival curves presenting six-year survival of restorations

The estimated mean survival time was 59.82 ± 1.50 months. For molars with mild MIH lesions, the survival probabilities were 99.2%, 95.9%, 89.8%, 82.5%, and 24.3%, respectively, while for molars with severe lesions, the probabilities were 98.1%, 91.7%, 85.9%, 75.6%, and 11.1% (Fig. 3). The estimated mean survival time for mild lesions was 62.68 ± 2.73 months, and for severe lesions, it was 58.85 ± 1.79 months. The results of the univariate and multivariate Cox proportional hazards regression analyses, calculated using robust standard errors, are presented in Table 3. According to the univariate analysis, neither age nor dental arch had a significant effect on restoration survival (*p* = 0.401 and *p* = 0.879, respectively). However, both lesion severity and lesion extension were found to significantly influence the hazard ratio for restoration survival (*p* < 0.001 and *p* < 0.001, respectively). In the multivariate analysis, lesion severity, medium lesion extension, and large lesion extension were each identified as significant predictors of restoration survival, with p-values below 0.001.

**Table 3:** Univariate and multivariate Cox regression comparisons of factors affecting restoration survival

Variable	Satisfied (*N* = 10)	Unsatisfied (*N* = 35)	Hazard ratio (95% Confidence Interval)	*p*-value*
Univariate comparison				
**Age**				
7–9 years	5 (17.9)	23 (82.1)	Reference	0.401
10–12 years	5 (29.4)	12 (70.6)	1.245 (0.815–1.902)	
**Dental Arch**				
Lower	4 (20)	16 (80)	Reference	0.879
Upper	6 (24)	19 (76)	1.472 (0.991–2.188)	
**Lesion Severity**				
Mild	5 (35.7)	9 (64.3)	Reference	**< 0.001**
Severe	5 (16.1)	26 (83.9)	0.734 (0.460–1.1172)	
**Lesion Extension**				
Small	5 (38.5)	8 (61.5)	Reference	**< 0.001**
Medium	4 (22.2)	14 (77.8)	0.587 (0.351–0.982)	
Large	1 (7.1)	13 (92.9)	0.626 (0.394–0.993)	
**Multivariate comparison**				
**Dental Arch**			1.442 (0.967–2.150)	0.535
**Lesion Severity**			1.025 (0.272–3.856)	**< 0.001**
**Lesion Extension (Medium)**			0.576 (0.140–2.362)	**< 0.001**
**Lesion Extension (Large)**			0.643 (0.400–1.032)	**< 0.001**
*Cox regression using with robust standard error to allow for clustering within patients. Bold values indicate significant difference; p < 0.05				

## Discussion

This study aimed to evaluate the clinical success and survival of glass hybrid restorations in permanent first molars affected by MIH following SCR over a six-year follow-up period. The findings indicated that the child’s age and the arch of the treated molar had no significant impact on restoration survival. However, when clustering within patients was accounted for using robust standard errors, lesion severity and lesion extension were found to significantly influence survival outcomes. Consequently, the primary null hypothesis was rejected. Meanwhile, the secondary null hypothesis, suggesting no significant differences in restoration satisfaction across the related evaluation categories, was accepted. Over the long-term follow-ups, a decline in the survival of glass hybrid restorations was observed, particularly in severely affected molars. This decline may be associated with the inherent durability limitations of glass ionomer-based materials, as well as changes in the mineral content and bonding strength of teeth affected by MIH.

When a molar affected by MIH exhibits post-eruptive enamel breakdown, atypical caries, or an incompatible existing restoration, selecting an appropriate restorative material becomes essential. This decision must consider the structural, chemical, and mechanical properties of MIH-affected enamel, along with the characteristics of the lesion [18]. Numerous studies in the literature have evaluated various restorative materials, including amalgams, composite resins, glass ionomer-based materials, preformed metal crowns, and laboratory-fabricated crowns. The latest guidelines from the EAPD [19] provide moderate evidence quality and strong recommendations for specific approaches: applying fissure sealants with adhesives for fully erupted mild cases, using composite resins under rubber dam isolation for mild/severe cases with invasive techniques, avoiding composite resins in minimally invasive approaches, employing self-etch or total-etch adhesive systems, deproteinizing with sodium hypochlorite before composite resin restorations, and applying preformed metal crowns [19]. In contrast, for minimally invasive techniques utilizing glass ionomer-based restorations, the evidence quality is moderate, and the recommendation strength is conditional [19]. This is primarily due to the limited number of studies examining glass ionomer-based restorations in MIH-affected molars and the relatively short follow-up periods in existing research. The present study extends previously reported findings by presenting six-year survival for restorations initially evaluated at two-year follow-ups [11]. As one of the few studies investigating the long-term success of glass ionomer-based restorations, this research provides valuable insights and holds a unique position in advancing the scientific understanding of MIH management.

In cases where a child is uncooperative for invasive treatments requiring local anesthesia or lacks access to routine dental care, glass ionomer-based restorations may be a preferred interim solution to protect the tooth from post-eruptive breakdown and hypersensitivity until a permanent restoration can be placed or extraction becomes appropriate for the child’s age [19]. As hydrophilic materials with fluoride release capability, glass ionomers are particularly advantageous in situations where ideal moisture control is unachievable. However, their primary limitation—low mechanical strength—restricts their use in stress-bearing areas [20]. The current study’s findings demonstrate a gradual decline in the survival of glass hybrid restorations, particularly in molars severely affected by MIH. This decline can be attributed to the mechanical properties of MIH-affected teeth and the inability of glass ionomers to withstand occlusal forces during mastication. Fragelli et al. [21] evaluated the 12-month survival rates of glass ionomer restorations in 48 MIH-affected molars, reporting a survival rate of 78% after one year. Their study highlighted the success of glass ionomers in single-surface defects and suggested these materials as a viable option due to their tooth-preserving properties and remineralization potential. In comparison, the current study documented survival probabilities of 95.5% at 6 months, 94% at 12 months, 87.5% at 18 months, and 87.5% at 24 months for glass hybrid restorations. Meanwhile, a retrospective cohort study reported cumulative survival probabilities of conventional glass ionomer restorations in small-to-medium-sized cavities as only 7% at 36 months [20]. Grossi et al. [22] reported a success rate of 93.3% at a 12-month follow-up for MIH-affected molars treated with atraumatic restorative treatment and glass hybrid restorations, concluding that both approaches were effective for preserving MIH-affected molars. Additionally, a split-mouth clinical study compared the success of glass hybrid restorations and short-fiber-reinforced composites following SCR. The 2-year survival were reported as 93.5% for short-fiber-reinforced composites and 77.4% for glass hybrid restorations [9]. Interestingly, the 3-year results from the same study indicated similar clinical success for both materials [12]. Variability in clinical findings among studies may result from differences in study designs, standardization of lesion severity and size, clinical conditions, operator experience, and the cooperation levels of treated children. Nevertheless, the current study corroborates previous research, indicating that glass hybrid restorations demonstrate acceptable survival rates and clinical performance over 2 years. However, their long-term survival declines, likely due to the specific properties of MIH-affected teeth and the mechanical limitations of the materials.

The results of the present study using multivariate Cox regression with robust standard error to account for clustering within patients have shown that lesion severity and extension have a significant effect on restoration survival. A clinical study, in which MIH-affected molars were restored with glass ionomer cement and followed for one-year, reported a significant correlation between increasing MIH severity and the extension of restorations to include two or more surfaces, with a 12-month survival rate of 78% for the affected teeth [21]. On the other hand, in a randomized clinical study by Rollim et al., which examined the survival of different adhesive restorations in MIH-affected molars, restorations involving cusp involvement were found to have a higher survival rate compared to those without, although the difference was not statistically significant [23]. In a longitudinal study investigating apical closure and restoration survival in teeth with open apices and affected by MIH, where glass ionomer and composite materials were applied after SCR, it was reported that the marginal integrity approach resulted in a 96.8% success rate for severely affected MIH teeth over a 24-month period [24]. However, the number of studies evaluating the long-term success of various treatment approaches and materials based on lesion severity and extension in MIH-affected molars is limited in the literature. In this context, the findings of the present study, which confirm the significant effect of lesion severity and extension on the survival of glass hybrid restorations, may provide guidance for further research and the clinical management of affected teeth.

Independent of MIH, several clinical studies have assessed the survival rates of glass hybrid restorations. A 60-month follow-up study comparing the success of glass hybrid and resin composite in non-carious cervical lesions among patients with bruxism reported no significant differences in survival rates between the two materials [25]. Similarly, a multicenter study evaluating glass hybrid and resin composite restorations in two-surface Class II cavities over a 5-year period found no significant differences in long-term survival rates [26]. Another study investigating the performance of these materials in large and deep Class II cavities also reported no significant differences in survival rates, except for the color match criterion, with both materials demonstrating satisfactory outcomes [27]. These findings suggest that similar to teeth unaffected by developmental dental anomalies, the survival of glass hybrid restorations in MIH-affected teeth is satisfactory for approximately two years on average. However, as noted earlier, the decline in survival rates during long-term follow-ups is likely due to the structural properties of MIH-affected enamel and the mechanical limitations of glass ionomers. An important consideration in these evaluations is the use of modified USPHS criteria, which may underestimate the true success rates of glass ionomer-based restorations. Expecting perfect performance in criteria such as color match, surface texture, or marginal discoloration from a glass ionomer-based material is unrealistic. In modern preventive dentistry, where minimally invasive approaches focus on creating cleanable surfaces and promoting remineralization, biological outcomes—such as the prevention of hypersensitivity, alleviation of the child’s complaints, and management of secondary caries—should be prioritized over aesthetic criteria. Although our findings were presented according to modified USPHS criteria, we believe that the actual success rates are higher when considered within the context of minimally invasive dentistry. This is supported by the rate of molars classified as unsatisfactory based on these criteria, but which were remineralized and symptom-free. To provide a more scientifically robust framework, we recommend that experts in the field work towards a consensus on developing a more comprehensive index system for evaluating the performance of glass ionomer-based restorations. Such an index would allow for more accurate and meaningful assessments of these materials in clinical practice.

Contemporary therapeutic approaches to carious lesions prioritize the preservation of pulp health in vital teeth with deep carious lesions [28]. To achieve this, SCR is emphasized, leaving the cavity margins hard while removing only the soft dentin overlying the pulp. The SCR technique, grounded in biological principles, aims to create a favorable microenvironment for dentin remineralization, maintain pulp vitality, ensure optimal marginal sealing, and arrest the progression of carious lesions [29]. In a study comparing the success of stainless-steel crowns and composite resins following SCR in MIH-affected molars, 2-year survival rates were reported as 94.4% for stainless steel crowns and 49.2% for composite resins [30]. The lower clinical success of composite resins was attributed to their limited remineralization capacity. In contrast, the high success rate of stainless-steel crowns was linked to their ability to completely isolate the tooth’s hard tissues from the oral environment. Similarly, studies evaluating the outcomes of glass hybrid restorations in MIH-affected teeth treated with SCR have demonstrated favorable results during 2-year follow-ups [9, 11]. The high remineralization potential of glass hybrid restorations has been credited for their effectiveness in alleviating hypersensitivity and arresting caries progression. These findings underscore the therapeutic potential of glass hybrid restorations as a reliable treatment option for managing MIH-affected molars, particularly when combined with the principles of SCR.

The present study has several limitations. Foremost among these is the inability to standardize cavity designs in MIH-affected molars and the inherent challenges in distinguishing between infected and affected tissues during SCR. Additionally, the absence of a control group represents a significant limitation. Another important limitation is the lack of any available data on patient follow-ups between the two-year and six-year follow-up periods. Despite these constraints, this study is notable as the first to report six-year follow-up outcomes of both glass hybrid restorations and SCR in MIH-affected molars. The extended follow-up period, coupled with the application of standardized treatment protocols and evaluations performed by experienced clinicians, is a key strength of this research.

Given the rising global prevalence of MIH, it remains a critical public health concern [31]. The unique characteristics of MIH-affected teeth impose significant limitations on the available treatment approaches. However, advancements in dental biomaterials and the adoption of minimally invasive dentistry provide promising opportunities for restoring and maintaining these teeth in a healthy state [32]. Future research should focus on standardizing cavity preparation protocols, developing durable restorative materials, and creating innovative indices for evaluating success beyond conventional criteria. These efforts will not only enhance long-term clinical outcomes but also address the growing global burden of MIH as an important oral health challenge.

## Conclusions

The findings indicate that while glass hybrid restorations demonstrate satisfactory survival in the short to medium term, their performance declines over time, particularly in severely affected molars by molar incisor hypomineralization. Despite these limitations, glass hybrid restorations remain a practical option for managing molar incisor hypomineralization-affected molars, especially in young patients where minimally invasive approaches are prioritized to preserve tooth structure.

This study underscores the critical role of material selection, lesion severity, lesion extension, and follow-up duration in determining clinical outcomes. When clustering within patients was accounted for using robust standard errors, lesion severity and lesion extension were found to significantly impact restoration survival. The notable decline in survival probability after six years, particularly in severely affected molars, highlights the urgent need for advancements in restorative materials and techniques. The long-term follow-up of restorations in molar incisor hypomineralization-affected teeth is essential to better understand their durability, particularly in the context of the challenges posed by these conditions. Future innovations should address the unique structural and chemical challenges posed by molar incisor hypomineralization-affected molars, aiming to improve long-term clinical success and patient outcomes.

## Data Availability

The data that support the findings of this study are available from the corresponding author (B.S.) upon reasonable requests.
